# Cryo-electron tomography reveals OmpB is required for the *Rickettsia* *parkeri* S-layer

**DOI:** 10.17912/micropub.biology.002219

**Published:** 2026-06-16

**Authors:** Meghan C. Bacher, Daniel Serwas, Yan Han, Dominika Borek, Matthew D. Welch

**Affiliations:** 1 Department of Molecular and Cell Biology, University of California, Berkeley, Berkeley, California, United States; 2 Biohub, Redwood City, California, United States; 3 Department of Biophysics, University of Texas Southwestern Medical Center, Dallas, Texas, United States; 4 Department of Biophysics, Department of Biochemistry, University of Texas Southwestern Medical Center, Dallas, Texas, United States

## Abstract

Many bacteria, and most archaea, have a paracrystalline protein surface layer (S-layer) encapsulating their outer membrane or cell wall. For pathogenic *Rickettsia* species, an S-layer has been hypothesized to be comprised of the outer membrane protein OmpB. We used cryo-electron tomography (cryoET) to image the *Rickettsia parkeri* cell surface and observed a repetitive S-layer structure proximal to the outer membrane of the bacterium that is absent in an *ompB ^STOP^* ::tn mutant. Our data reveal that OmpB is essential for the *Rickettsia* S-layer and suggest that we can leverage cryoET to examine rickettsial S-layer structure and function.

**Figure 1. The surface of R. parkeri is encapsulated by an S-layer that is absent in ompB STOP ::tn mutant bacteria:** (A) AlphaFold 3 protein folding prediction of *R. parkeri* OmpB, color coded according to AlphaFold estimated pLDDT values (Abramson et al., 2024). (B) Tomographic slices of the surface of wild-type and *ompB ^STOP^* ::tn *R. parkeri* bacteria in A549 human epithelial cells at 24 hpi *.* Scale bar = 200 nm. (i and ii) Insets represent magnified views of the regions of the bacterial surface indicated in the white boxes. Arrowheads indicate the S-layer in purple, outer membrane in blue, and inner membrane in green. Scale bar = 50 nm. (C) Tomographic slices of isolated wild-type and *ompB ^STOP^* ::tn *R. parkeri* bacteria, paired with segmentation models of the bacterial membranes and S-layer in the XY and YZ planes color coded as in (B). (D) A tomographic slice from cryo-FIB-milled wild-type *R. parkeri* paired with a segmentation model of the bacterial membranes and S-layer in the XY plane. Scale bar = 100 nm. (iii) Expanded view of the bacterial surface indicated in the white box with arrowheads color coded as in (B). Purple asterisks denote examples of the slanted S-layer architecture. Scale bar = 20 nm.

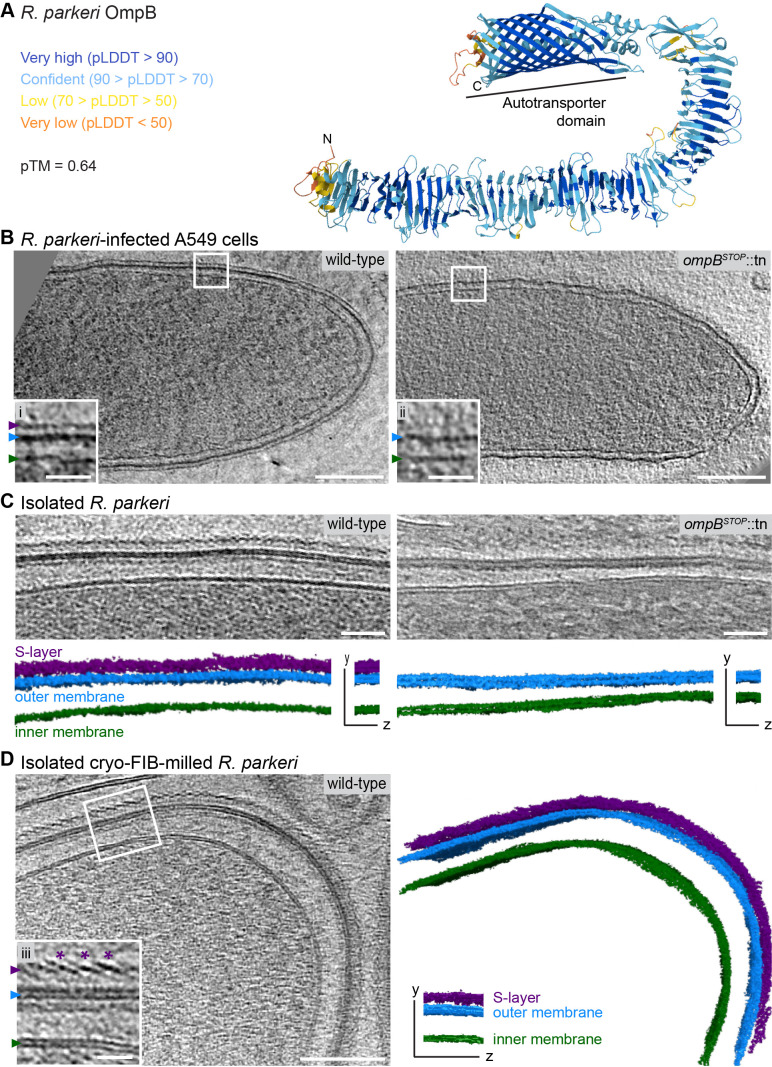



## Description

The *Rickettsia* genus comprises numerous species of arthropod-associated obligate-intracellular Gram-negative alphaproteobacteria, some of which are human pathogens causing various forms of rickettsiosis (Blanton, 2019). *Rickettsia parkeri* , which is endemic to the Americas and causes an eschar-associated spotted fever rickettsiosis (Paddock et al., 2004; Paddock et al., 2008), has emerged as a model for studying rickettsial infections. These bacteria invade host cells, escape from a membrane-bound phagosome, and grow within the host cell cytosol. Early electron microscopy images of various *Rickettsia* species noted the presence of a paracrystalline surface layer (S-layer) enmeshing the bacteria (Palmer et al., 1974; Popov and Ignatovich, 1976; Silverman et al., 1978). S-layers, which are found on most archaea and some bacteria, are ordered lattices that are formed by one or more S-layer proteins (Assandri et al., 2023; Grill-Walcher and Schäffer, 2025; Isbilir et al., 2026). Functions of the S-layer include membrane stabilization, microbe-microbe adhesion, and protection from phage infection (Assandri et al., 2023; Grill-Walcher and Schäffer, 2025). A high-resolution structure of the S-layer was determined for the related alphaproteobacterium *Caulobacter crescentus* using a combination of X-ray crystallography of the purified S-layer protein RsaA and cryo-electron tomography (cryoET) of intact bacteria (Bharat et al., 2017). The RsaA protomers fold into Ca ^2+^ -coordinating β-helices that oligomerize to form a hexameric lattice (Bharat et al., 2017) that attaches to the LPS O-antigen on the surface of the bacterium (Awram and Smit, 2001; von Kügelgen et al., 2020). These studies provide a framework to investigate the structure of other alphaproteobacterial S-layers by cryoET.

The *Rickettsia* spp. S-layer has been proposed to be composed primarily of the outer membrane protein OmpB (Ching et al., 1990; Ching et al., 1996), which comprises approximately 10% of the total protein mass (Dasch et al., 1981). OmpB was originally characterized as a surface antigen (Dasch et al., 1981) and was subsequently shown to participate in host cell invasion (Chan et al., 2009) as well as protection from ubiquitylation and subsequent autophagy by the host cell (Engström et al., 2019; Engström et al., 2021). OmpB is conserved in *Rickettsia* species (Lehman et al., 2024) and is a member of the type V secretion system or autotransporter protein family (Blanc et al., 2005), consisting of an N-terminal passenger domain and C-terminal autotransporter domain. After translocation to the cell surface, OmpB is cleaved (Carl et al., 1990), leaving a 120 kDa fragment that is postulated to multimerize on the bacterial surface (Ching et al., 1990). Using AlphaFold 3 (Abramson et al., 2024), the structure of the passenger domain *R. parkeri* OmpB protein is predicted to fold into a series of β-sheets that emanate from the membrane-anchored autotransporter domain ( Figure 1A ), similar to the secondary structures in the *C. crescentus* RsaA protein (Bharat et al., 2017). However, whether OmpB is critical for the formation of the S-layer on the rickettsial surface has not been determined.

We imaged the bacterial surface by cryoET, first in an infected mammalian cell line, as the organism is obligately intracellular and thus best examined in its native context. Human A549 lung epithelial cells grown on holey carbon grids were infected with *R. parkeri* , and at 24 h post infection (hpi), were permeabilized for 30 s in the presence of both 0.5% Triton X-100 to facilitate cryoET of unmilled infected cells by removing cytoplasmic host cell material to reduce background noise, and 0.25% glutaraldehyde to gently fix and preserve protein structures (Urban et al., 2010; Mueller et al., 2014; Fäßler et al., 2020). Samples were then further fixed with 2% glutaraldehyde for at least 15 min and subsequently plunge frozen. CryoET imaging was carried out, targeting tilt series collection to thin regions of infected cells near the cell periphery. In tomograms containing bacteria *,* the inner and outer bacterial membranes were clearly visible, along with the S-layer surrounding the outer membrane (67 tomograms, 4 grids) ( Figure 1B ). To test whether OmpB is required to form the outer S-layer, we infected cells with the *R. parkeri* *ompB ^STOP^* ::tn mutant, carrying both a nonsense mutation and transposon insertion in the *ompB* gene that minimally impact internalization of bacteria into hosts cells (Engström et al., 2019; Engström et al., 2021), and processed for imaging as described above. In tomograms of *R. parkeri ompB ^STOP^* ::tn mutant bacteria, the inner and outer bacterial membranes were visible, but the outermost electron-dense S-layer was absent (16 tomograms, 1 grid) ( Figure 1B ). This indicates that OmpB is essential for S-layer formation and provides further evidence that OmpB is a major structural component of this layer.

To verify that permeabilization and fixation did not alter the presence of the S-layer, we purified bacteria from host cells, spotted them onto holey carbon grids, proceeded directly to plunge freezing without permeabilization or fixation, and then imaged using cryoET ( Figure 1C ). In these unfixed samples, the bacterial inner membrane appeared to be straighter, perhaps because permeabilization and fixation affected membrane structure. Moreover, the periplasmic space (distance between the inner and outer bacterial membranes) appeared to be larger and more variable in size, perhaps because isolation of bacteria from the host cell environment caused a stress response. Nevertheless, in tomograms of isolated bacteria, the S-layer was again clearly visible on the surface of wild-type *R. parkeri* (34 tomograms, 1 grid), but absent in the *ompB ^STOP^* ::tn mutant (47 tomograms, 1 grid) ( Figure 1C ).

To obtain higher-resolution tomograms of the bacterial surface, purified bacteria were further thinned through cryo-focused ion beam (cryo-FIB) milling (Khanna and Villa, 2022) after being spotted and plunge frozen on holey carbon grids (without permeabilization or fixation). Briefly, a gallium ion beam was used to mill away material from the top and bottom of the frozen bacterial lawn sample to a final thickness of about 200 nm. Following FIB-milling and cryoET imaging, the S-layer was better resolved, exhibiting a repetitive slanted lattice-like architecture ( Figure 1D ). We analyzed all tomograms comprising the cryo-FIB-milled dataset (22 tomograms, 2 grids) and found the external edge of the S-layer density is located an average of 14 ± 2 nm (SD) from the *R. parkeri* outer membrane, in the range of what was found for the S-layer or microcapsular layer in various negatively stained *Rickettsia* spp. samples (7-16 nm) (Palmer et al., 1974; Silverman et al., 1978) and slightly closer to the outer membrane than for *C. crescentus* (18-23 nm) (Bharat et al., 2017).

This study demonstrates the power of using cryoET to observe the *R. parkeri* surface. We confirmed the presence of an S-layer and demonstrated that the S-layer requires OmpB. Higher-resolution analysis of the OmpB structure and its association with other surface proteins and LPS will require the acquisition and subtomogram averaging of higher magnification tomograms of the bacterial surface. In addition to OmpB, the *Rickettsia* S-layer is proposed to contain OmpA (Ching et al., 1990; Ching et al., 1996), a larger and less abundant protein (OmpA:OmpB molar ratio is 1:9) (Policastro and Hackstadt, 1994). Similar to OmpB, OmpA is predicted to fold into a series of β-sheets, and the loss of OmpB may disrupt OmpA within the S-layer, compromising the lattice-like structure. Further structural analysis will be informative for determining how OmpB may interact with OmpA within the S-layer, as well as how OmpB shields OmpA and other surface proteins from ubiquitylation (Engström et al., 2019; Engström et al., 2021). This type of analysis will also be informative for understanding how the structure of the rickettsial S-layer compares with that of other microbes.

## Methods

**Mammalian cell culture**

Vero (ATCC Cat# CCL-81, RRID:CVCL_0059) and A549 (ATCC Cat# CRM-CCL-185, RRID:CVCL_0023) cells were sourced from the University of California, Berkeley, Cell Culture Facility. Vero cells were grown in DMEM containing 4.5 g/L D-glucose, 4 mM L-glutamine (Gibco, 11965118), and 2% fetal bovine serum (Gemini Bio-products, 100-500). A549 cells were grown at 37 ˚C and 5% CO _2_ in DMEM containing 4.5 g/L D-glucose, 4 mM L-glutamine (Gibco, 11965118), and 10% fetal bovine serum (ATLAS Biologics, FP-0500-A).

***Rickettsia* strain construction and propagation**

*R. parkeri* strain Portsmouth was obtained from Chris Paddock at the Centers for Disease Control and Prevention. Fluorescent *R. parkeri* harboring pRAM18dR-GFPAa (Figueroa-Cuilan et al., 2023) were generated as described previously (Bacher et al., 2026). Briefly, a T75 flask of Vero cells infected with *R. parkeri* was harvested, resuspended in K36 buffer (50 mM KH _2_ PO _4_ , 50 mM K _2_ HPO _4_ , pH 7.0, 100 mM KCl, 15 mM NaCl), and broken open with 1 mm beads by two 30 s rounds of vortexing. Cell debris was pelleted at 10,000 rpm for 2 min at 4 ˚C, and the supernatant was washed 3× with 250 mM sucrose. Washed bacteria were resuspended in 100 µL brain heart infusion (BHI) media (BD Difco, DF0418-17-7) and incubated on ice for 20 min with 10 µg of the pRAM18dR-GFPAa plasmid. Bacteria were electroporated in a 0.2 cm cuvette (Bio-Rad, 165-2086) at 2.5 kV, 200 Ω, 25 µF, resuspended in 1.2 mL BHI media, and added to a new T75 flask of Vero cells. The next day, 500 ng/mL rifampicin (Sigma, R3501; resuspended to 1 mg/mL in methanol and filter sterilized) was added to the infected cells. Fluorescent *Rickettsia* were harvested from infected Vero cells by adding cold sterile water for 2 min to break bacteria out of the cells. Freed bacteria were pelleted, resuspended in BHI media, and frozen at -80 ˚C.

*R. parkeri* GFPAa (wild-type) and *R. parkeri ompB ^STOP^* ::tn (Engström et al., 2019) bacteria were propagated and stored as a 30% preparation stock as described previously (Bacher et al., 2026). Briefly, *Rickettsia* bacteria were allowed to infect five confluent T175 flasks of Vero cells for 4-7 d at 33 ˚C. Infected cells were harvested, pelleted, and resuspended in 10 mL of K36 buffer. Bacteria were released from Vero cells by Dounce homogenization and isolated by centrifugation in a solution of 30% RenoCal-76 (Bracco Diagnostics, 04H208) in K36 buffer using a SW-32 Ti rotor (Beckman/Coulter) at 18,500 rpm for 30 min at 4 ˚C. The bacterial pellet was resuspended in 2 mL BHI media and aliquoted before freezing at -80 ˚C.

**Preparation of EM grids with host cells infected with *R. parkeri***

Holey carbon grids (Quantifoil R2/1, 200 mesh, gold, SPI, 4320G-FA) were cleaned by treating with acetone for 20 min, blotted with Whatman No. 1 filter paper, and then treated with 70% ethanol for 20 min. For *ompB ^STOP^* ::tn samples, grids were placed into 6-well plates with A549 cell culture media and incubated at 37 ˚C overnight. For wild-type samples, grids were placed into the wells of a 6-well plate with sterile water and then incubated in 0.1% poly-L-lysine (Sigma, P-8920) for 15 min, and washed 2× with 1× PBS (Gibco, 10010-023) before A549 cell culture media was added for 1-24 h. Incorporation of a poly-L-lysine coating during grid preparation increased A549 cell adhesion and the total number of infected cells per grid. Once grids were primed in media, fresh A549 media was added to the wells with grids, A549 cells were dripped onto the grids, and seeded grids were incubated overnight at 37 ˚C in 5% CO _2_ . The next day, *R. parkeri* GFPAa or *R. parkeri* *ompB ^STOP^* ::tn (MOI of 1-10) in cell culture media were added and grids were centrifuged at 300× g for 5 min. Infected cells were incubated for 24 h at 33 ˚C in 5% CO _2_ and then fixed as described below and previously (Urban et al., 2010; Mueller et al., 2014; Fäßler et al., 2020).

For fixation, grids were transferred to a drop of 0.5% Triton X-100 (Sigma, T9284), 0.25% glutaraldehyde (EMS, 16120), and cytoskeleton buffer (10 mM MES, pH 6.8, 150 mM NaCl, 5 mM EGTA, 5 mM glucose, 5 mM MgCl _2_ ) on parafilm for 30 s to permeabilize host cells. Grids were then moved to a drop of liquid containing 1 µg/mL unlabeled phalloidin (Sigma, P2141) or Alexa 568 phalloidin (Molecular Probes, A12380), 2% glutaraldehyde (EMS, 16120), and cytoskeleton buffer for at least 15 min for further fixation. Phalloidin was included to visualize bacterial actin tail structures, which is outside the scope of this study.

Post fixation, 7 µL of 10 nm gold beads (EMS, 25487, 6× concentrated) were pipetted onto the grids, the grids were blotted with Whatman No. 1 paper, and then another 3 µL of gold beads was added. Gold beads were used as fiducials for tomogram reconstruction. The grids were plunge-frozen in liquid ethane using an EM GP2 plunge freezer (Leica) after a 4-7 s blot. Grids were stored in liquid nitrogen until further use.

**Preparation of EM grids with a bacterial lawn**

Grids were prepared using a protocol adapted from Khanna and Welch, 2024. Holey carbon grids (Quantifoil R2/1, 200 mesh, copper, EMS, Q2100CR1; Quantifoil R2/1, 200 mesh, gold, SPI, 4320G-FA; or Quantifoil R1/2, 200 mesh, gold, EMS, Q2100AR-12,) were glow discharged for 10 s at 15 mA using a Pelco easiGlow system (RRID:SCR_020396). A total of 7 µL of *R. parkeri* GFPAa or *R. parkeri* *ompB ^STOP^* ::tn bacteria (3.5-5×10 ^7^ bacteria) from a frozen 30% preparation stock were spotted onto a grid as follows. Half (3.5 µL) of the bacterial volume was pipetted, the grid was blotted with Whatman No. 1 filter paper, and then the other half of the bacterial volume (3.5 µL) was added. The grids were plunge-frozen in liquid ethane using an EM GP2 plunge freezer (Leica) after a 4-7 s blot. Grids were stored in liquid nitrogen until further use.

For cryo-FIB-milling, grids were transferred to the Stanford-SLAC CryoET Specimen Preparation Center (SCSC) and loaded into an Aquilos 2 Cryo-FIB microscope (ThermoFisher Scientific, RRID:SCR_019880) for milling with a 30 kV focused ion beam. Grids were sputter coated with metallic platinum, coated with organometallic platinum using a gas injection system for 1 min, and then sputter-coated again with metallic platinum. AutoTEM 5 software (ThermoFisher) was used to determine the milling angle for all lamella sites. For some samples, the AutoTEM 5 software (ThermoFisher) was used to completely mill lamellae to a thickness of 200 nm. Incompletely milled lamellae were manually milled with 500 pA (rough milling) that was gradually reduced to 30 pA (polishing) to obtain 200 nm thick lamellae. Stress relief cuts were included to limit lamellae breakage.

**Tilt series collection and processing**

For the *ompB ^STOP^* ::tn sample in Figure 1B, tilt series were collected at UC Berkeley CalCryo@QB3 facility using a Titan Krios (FEI) operated at 300 kV and equipped with a K2 direct electron detection device (Gatan) and quantum energy filter (Gatan). Images were collected at a 3.36 Å pixel size and with a target defocus of -6 µm. Automated tilt series acquisition was performed using Serial EM v4.0.12 (Mastronarde, 2005) using a bidirectional tilt scheme with an increment of 2˚ and a total electron dose of 120 e ^-^ /Å ^2^ .

For Figures 1B wild-type, 1C, and 1D, tilt series were collected at the UW Madison Cryo-EM Research Center Midwest Center for Cryo-Electron Tomography (MCCET). Tilt series were acquired on a Titan Krios (ThermoFisher) at 300 kV equipped with a K3 electron detection device (Gatan) and quantum energy filter (Gatan) using Serial EM v4.1.0 (Mastronarde, 2005).

For Figure 1B, wild-type, automated tilt series were acquired at a pixel size of 3.7 Å and target defocus of -6 µm using a dose-symmetric tilt scheme with increments of 3˚ and a total electron dose of 120 e ^-^ /Å ^2^ . For Figure 1C, wild-type, automated tilt series were acquired at a pixel size of 3 Å and target defocus of -4 to -6 µm using a dose-symmetric tilt scheme with increments of 3˚ and a total electron dose of 120 e ^-^ /Å ^2^ . For Figure 1C, *ompB ^STOP^* ::tn *,* automated tilt series were acquired at a pixel size of 1.9 Å and target defocus of -6 µm using a dose-symmetric tilt scheme with increments of 3˚ and a total electron dose of 100 e ^-^ /Å ^2^ . For Figure 1D, automated tilt series were acquired at a pixel size of 2.4 Å and target defocus of -3 to -5 µm using a dose-symmetric tilt scheme with increments of 3˚ and a total electron dose of 100 e ^-^ /Å ^2^ .

For initial screening of tomograms, tilt series were motion corrected with MotionCor2 1.6.4 (Zheng et al., 2017) and then aligned and reconstructed using AreTomo2 v1.1.2 (Zheng et al., 2022). For final reconstruction of tomograms with fixed infected cells, tilt series were aligned in IMOD v5.1 (Kremer et al., 1996) (RRID:SCR_003297) using the 10 nm gold beads as fiducials for alignment and weighted back-projection algorithm with a simultaneous iterative reconstruction technique (SIRT)-like filter. Tomograms were binned by a factor of 3 and then processed with the nonlinear anisotropic diffusion (NAD) filter. For final reconstruction and visualization of tomograms without gold fiducials, tilt series were motion-corrected using Relion 5.0 and aligned using AreTomo2 default parameters through the wrapper within Relion 5.0 (Burt et al., 2024) (RRID:SCR_016274). Contrast transfer function (CTF) estimation was also performed with AreTomo2 during tilt series alignment. Tomogram reconstruction was performed in Relion 5.0 after removal of dark images and tomograms were binned to a pixel size of 10 Å.

**Tomogram segmentation and S-layer quantification**

For Figures 1C and 1D, manual segmentation of 27-75 slices of representative wild-type and *ompB ^STOP^* ::tntomograms was performed in Dragonfly 3D (v2025.1; Comet Technologies Inc.). The S-layer thickness (edge of outer membrane density to outer edge of S-layer density) of wild-type FIB-milled bacteria was measured at two locations per tomogram using the contour modeling function in IMOD v4.11.25 (Kremer et al., 1996).

**Data availability**

Tomograms have been deposited to the Biohub CryoET Data Portal database under deposition code: CZCDP-10353.

## Reagents

**Table:** 

**Strain**	**Genotype**	**Reference**
*R. parkeri* GFPAa (wild-type)	*R. parkeri* strain Portsmouth harboring the pRAM18dR-GFPAa shuttle vector	This study
* R. parkeri ompB ^STOP^* ::tn	*ompB* null *R. parkeri* strain Portsmouth harboring both a nonsense mutation and a transposon insertion in the *ompB* gene	Engström et al., 2019
**Plasmid**	**Genotype**	**Description**
GFPAa	pRAM18dR-GFPAa	GFPuv in pRAM19dRGA is replaced with AausFP1. From Figueroa-Cuilan et al., 2023.
